# Gene Expression Profile of the Cerebral Cortex of Niemann-Pick Disease Type C Mutant Mice

**DOI:** 10.3390/genes16080865

**Published:** 2025-07-24

**Authors:** Iris Valeria Servín-Muñoz, Daniel Ortuño-Sahagún, María Paulina Reyes-Mata, Christian Griñán-Ferré, Mercè Pallàs, Celia González-Castillo

**Affiliations:** 1 Laboratorio de Neuroinmunobiología Molecular, Instituto de Neurociencias Translacionales (INT), Departamento de Biología Molecular y Genómica, Centro Universitario de Ciencias de la Salud (CUCS), Universidad de Guadalajara, Sierra Mojada 950, Guadalajara 44340, Mexico; iris.servin@alumnos.udg.mx; 2 Doctorado en Ciencias Biomédicas, Centro Universitario de Ciencias de la Salud (CUCS), Universidad de Guadalajara, Guadalajara 44340, Mexico; 3 Departamento de Disciplinas Filosófico, Metodológicas e Instrumentales, Centro Universitario de Ciencias de la Salud (CUCS), Universidad de Guadalajara, Guadalajara 44340, Mexico; paulina.reyes@academicos.udg.mx; 4 Pharmacology Section, Department of Pharmacology, Toxicology and Therapeutic Chemistry, Faculty of Pharmacy and Food Sciences, Institute of Neuroscience, Universitat de Barcelona, 08028 Barcelona, Spain; christian.grinan@ub.edu (C.G.-F.); pallas@ub.edu (M.P.); 5 Centro de Investigación Biomédica en Red (CiberNed), Network Center for Neurodegenerative Diseases, National Spanish Health Institute Carlos III, 28220 Madrid, Spain; 6 Tecnologico de Monterrey, Escuela de Medicina y Ciencias de la Salud, Campus Guadalajara, Zapopan 45201, Mexico

**Keywords:** NPC, Niemann Pick Type C, microarray, metabolism 1C, cortical circadian rhythm, ubiquitination, proteostasis

## Abstract

Background/Objectives: Niemann-Pick disease Type C (NPC) represents an autosomal recessive disorder with an incidence rate of 1 in 100,000 live births that belongs to the lysosomal storage diseases (LSDs). NPC is characterized by the abnormal accumulation of unesterified cholesterol, in addition to being an autosomal recessive inherited pathology, which belongs to LSDs. It occurs in 95% of cases due to mutations in the NPC1 gene, while 5% of cases are due to mutations in the NPC2 gene. In the cerebral cortex (CC), the disease shows lipid inclusions, increased cholesterol and multiple sphingolipids in neuronal membranes, and protein aggregates such as hyperphosphorylated tau, α-Synuclein, TDP-43, and β-amyloid peptide. Mitochondrial damage and oxidative stress are some alterations at the cellular level in NPC. Therefore, the aim of this work was to determine the gene expression profile in the CC of NPC1 mice in order to identify altered molecular pathways that may be related to the pathophysiology of the disease. Methods: In this study, we performed a microarray analysis of a 22,000-gene chip from the cerebral cortex of an NPC mutant mouse compared to a WT mouse. Subsequently, we performed a bioinformatic analysis in which we found groups of dysregulated genes, and their expression was corroborated by qPCR. Finally, we performed Western blotting to determine the expression of proteins probably dysregulated. Results: We found groups of dysregulated genes in the cerebral cortex of the NPC mouse involved in the ubiquitination, fatty acid metabolism, differentiation and development, and underexpression in genes with mitochondrial functions, which could be involved in intrinsic apoptosis reported in NPC, in addition, we found a generalized deregulation in the cortical circadian rhythm pathway, which could be related to the depressive behavior that has even been reported in NPC patients. Conclusions: Recognizing that there are changes in the expression of genes related to ubiquitination, mitochondrial functions, and cortical circadian rhythm in the NPC mutant mouse lays the basis for targeting treatments to new potential therapeutic targets.

## 1. Introduction

Niemann-Pick disease Type C (NPC) has a low incidence worldwide, estimated at approximately 1:100,000 cases per birth [1]. It is a disease that belongs to the group of lysosomal storage diseases (LSDs) and is characterized by the accumulation of compounds in the lysosomes. An accumulation of non-esterified cholesterol mainly characterizes NPC, but also sphingomyelin, sphingosine, and gangliosides (GM2 and GM3).

NPC is an autosomal recessive pathology caused in 95% of cases by mutations in the NPC1 gene, while 5% of cases are due to mutations in the NPC2 gene. These genes encode the proteins NPC1 and NPC2, which are involved in the intracellular transport of cholesterol and mediate its release from the endosome–lysosome system [2,3]. The accumulation of cholesterol occurs in various tissues, with the liver and CNS being the most affected [4].

In the cerebral cortex, the disease shows lipid inclusions, increased cholesterol and various sphingolipids in neuronal membranes, and protein aggregates such as hyperphosphorylated tau, α-Synuclein, TDP-43, and β-amyloid peptide [5]. In NPC, some changes have been reported at the cellular level, such as mitochondrial damage, oxidative stress, intrinsic apoptosis, and increased autophagy [6,7]. However, few high-resolution expression studies allow us to learn more about the molecular mechanisms that are altered in this pathology, and currently, there are no studies in the cerebral cortex that allow us to elucidate altered mechanisms that cause protein aggregation, oxidative stress, and intrinsic apoptosis.

The Npc1imagine mice model was generated by inserting a human intronic mutation (c.1554-1009G>A) into mouse intron 9, resulting in a pseudoexon that alters splicing and causes a truncated Npc1 protein. This model has characteristic features of NPC, such as an accumulation of cholesterol, GM2 and GM3 gangliosides, dihydroceramide, and neurological effects visible from 7 to 8 weeks [8].

In this work, we performed a microarray expression analysis to determine the expression of 22,000 genes in the cerebral cortex of NPC mice compared to WT mice and detected alterations in genes involved in functions of ubiquitination, mitochondrial metabolism, apoptosis, differentiation and development, and fatty acid metabolism.

## 2. Materials and Methods

### 2.1. Animals

The mice have a C57BL/6 genetic background and were kindly provided by the Addi and Cassi Fund for this study (http://addiandcassi.com/) after being generated by Ozgene. The crosses were performed according to the protocol by Gomez Grau et al. The heterozygous Npc1imagine/+ mice were crossed and resulted in litters consisting of Npc1imagine/imagine, Npc1imagine/+, and Npc1+/+. Npc1imagine/imagine mice were used as NPC mutant mice, and Npc1+/+ mice are the wild-type mice. Animals had free access to food and water and were maintained under standard temperature conditions (22 ± 2 °C), relative humidity between 40 and 50%, and a 12 h:12 h light/dark cycle (300 lux/0 lux). Every effort was made to reduce the number of animals and their suffering.

Male mice were used for all experiments. Animals were randomly assigned to experiments using the following numbers per experiment: for microarray and Western blot for the WT group (*n* = 4) and the NPC (*n* = 4) and for qPCR for the WT group (*n* = 6) and the NPC (*n* = 7). No sample size calculation was performed. Only male mice were used in this study to avoid possible hormonal fluctuations due to the estrus cycle of female mice.

### 2.2. Preparation of the Tissue

The mice were sacrificed at 60 days by a lethal injection of pentobarbital (60 mg/kg, i.p.) and then decapitated. The cerebral cortex was removed and stored in RNAlater (ThermoFisher, Waltham, MA, USA, catalog #AM720) at −80 °C until processing. Mice were sacrificed at the same time during the day.

### 2.3. RNA Extraction and cDNA Synthesis

The whole cerebral cortex was homogenized with a pellet pestle, 600 μL of lysis buffer from the Purelink^®^ RNA mini kit (Ambion #10359103) was added, and RNA extraction was performed according to this kit’s instructions. Purity and quality were determined spectrophotometrically using a NanoDrop™ ND-2000 (Thermo Scientific, Waltham, MA, USA). The cDNA (complementary DNA) synthesis was performed with the script cDNA Synthesis iScriptTM kit (#1708891, Bio-Rad Laboratories, Hercules, CA, USA) using the required amount of RNA to obtain 300 ng/μL.

### 2.4. Microarray Analysis

cDNA was synthesized from 10 μg of extracted total RNA and double-labeled with the fluorescent markers dUTP-Cy3 and dUTP-Cy5. cDNA was hybridized to a chip containing 22,000 oligo mouse arrays as previously described [9,10,11]. The quantification of array images was performed using the ScanArray 4000 (Packard BioChips Technologies; Billerica, MA, USA), and mean Cy3 and Cy5 background values were calculated using ArrayPro Analyzer software version 6.3 (Media Cibernetics, Rockville, MD, USA). Microarray data were analyzed using GenArise software version 3.18, developed by the Computer Department of the Institute of Cell Physiology of UNAM [12,13]. This software allows the identification of differentially expressed genes (DEGs) by calculating the Z-Score value, which indicates the number of standard deviations by which each gene deviates from its mean value. It uses a sliding window algorithm to determine the mean and standard deviation within a given window around each data point and generate a Z-Score that reflects the distance of a data point from the mean in standard deviation units. Based on the genArise analysis, genes that have a Z-Score of ±1.5 are considered statistically significant for differential expression (*p* < 0.05) [12,13]. To increase rigor, we established two additional, more restrictive Z-score thresholds for subsequent analyses, following previous studies [9,10,11], as described in the Bioinformatics section below.

### 2.5. Bioinformatic Analysis

For the bioinformatic analysis, a functional analysis of the microarray results, as well as a cluster and KEGG (Kyoto Encyclopedia of Genes and Genomes) pathway analysis, were performed. Cluster analysis was performed using Functional Annotation Clustering, available through DAVID Bioinformatics [14] https://david.ncifcrf.gov/home.jsp (accessed 29 April 2025). KEGG pathway analysis was performed in the databases DAVID, Genetrail (GenetrailV3.2), and (ShinyGO V0.77), considering genes with a Z-Score greater than ±2 for Cluster and KEGG analysis. Genes with a Z-score greater than 2.0 were selected for cluster analysis using the Database for Annotation, Visualization, and Integrated Discovery (DAVID) Bioinformatics Resources v.6.8 [14], which allows efficient organization of extensive gene lists into functionally related groups.

For the functional analysis, a more stringent threshold of Z-score ≥ 3.0 was applied to identify genes with the highest probability of differential expression, enabling individual gene analysis with Mouse Genome Informatics (MGIs) and the validation of microarray results by qPCR. This Z-score threshold of 3.0 is supported by our previous experimental results [9,10,11]. The microarray data were entered into the GEO database in accordance with the MIAME (Minimum Information About a Microarray Experiment) and MINSEQE (Minimum Information About a Next-generation Sequencing Experiment) guidelines

### 2.6. Gene Expression by qPCR

The qPCR (quantitative polymerase chain reaction) was performed on the StepOnePlus instrument with TaqMan probes (Applied Biosystems, Foster, CA, USA). The reaction was performed under the conditions indicated for the use of the master mix: 2 min of initiation at 50 °C, followed by 40 cycles consisting of a 20-s denaturation at 95 °C and an anneal and extension at 60 °C for 30 s. Data were analyzed by the comparative cycling threshold (Ct) method (ΔΔCt) using the glyceraldehyde-3-phosphate dehydrogenase (GAPDH) transcript as a constitutive control for normalization.

### 2.7. Determination of Protein Content by Western Blotting

The protein concentration was determined using the Bradford method. In total, 15 μg of protein samples were separated by sodium dodecyl sulfate-polyacrylamide gel electrophoresis (SDS-PAGE) (12%) and transferred to polyvinylidene difluoride (PVDF) membranes (#IPVH00010 Millipore). The membranes were blocked in 5% fetal bovine serum (FBS) in Tris-buffered saline (TBS) containing 0.1% Tween-20 (TBS-T) for 1 h at room temperature, followed by incubation with the primary antibodies overnight at 4 °C. The membranes were then washed and incubated with secondary antibodies for 1 h at room temperature. Immunoreactive proteins were visualized using the chemiluminescence detection kit according to the manufacturer’s protocol (ECL Kit; Millipore, Billerica, MA, USA), and digital images were captured using the ChemiDoc XRS+ system (BioRad, Hercules, CA, USA). Semiquantitative analyses were performed using ImageLab software (BioRad, Hercules, CA, USA) with GAPDH as a normalizing protein expression.

### 2.8. Statistical Analysis

Data analysis was performed using GraphPad Prism ver. 9.5 statistical Software. Data are expressed as mean ± standard error of the mean (SEM). All data were tested for normal distribution using the Shapiro–Wilk normality test. A comparison between groups was also performed using a two-tailed Student’s *t*-test for independent samples and a U Mann–Whitney test when necessary. Statistical significance was assumed if *p*-values were <0.05. Statistical outliers were identified using the Rout test and removed from the analysis if necessary.

## 3. Results

In the microarray analysis of 22,000 genes in the cerebral cortex of the NPC mutant mouse compared to the WT mouse, differentially expressed genes (DEGs) with a Z-Score value greater than 2 or less than −2 were selected. In this way, 952 genes were selected, of which 611 were found to be underexpressed and 341 were found to be overexpressed, with a tendency for down-regulation in the NPC mouse.

### 3.1. Functional and Cluster Classification of DEGs in the Cerebral Cortex of the NPC Mouse Mutant

**Figure 1:**
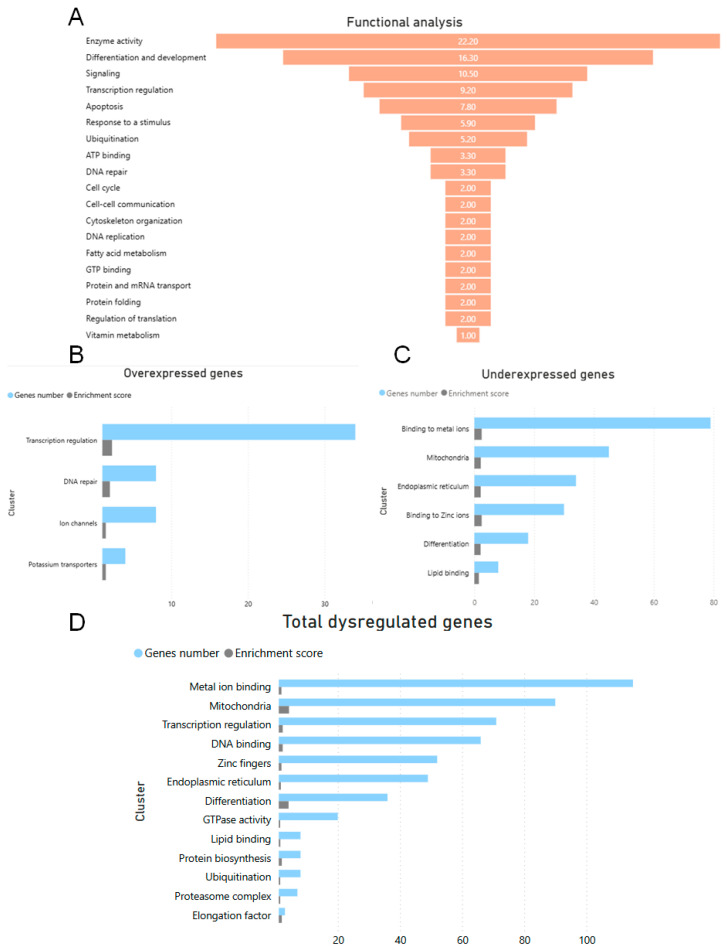
Functional and cluster classification of DEGs. (**A**) Functional analysis of differentially expressed genes (DEGs) with a Z-Score greater than ±3.0 in the cerebral cortex of the NPC mouse compared to the WT mouse. This analysis was performed on a total of 149 genes, of which 29 were overexpressed and 120 were underexpressed and classified according to the biological function in which they are involved. This mainly shows changes in genes involved in enzymatic activity but also changes in other important functions such as ubiquitination and apoptosis. Information for this analysis was obtained from Mouse Genome Informatics (MGI) https://www.informatics.jax.org/ (accessed 29 April 2024). FAs: Fatty acids. (**B**) Cluster analysis of overexpressed genes shows changes in groups of genes involved in metal ion binding, mitochondria, endoplasmic reticulum, and differentiation. (**C**) The clusters of overexpressed genes show significant genes involved in transcriptional regulation and DNA repair. (**D**) The gene clusters that remain significant in the total number of genes are transcriptional regulation, mitochondria, metal ion binding, and lipid binding. Cluster analysis of total differentially expressed genes with a Z-Score < −2 and >2 using DAVID Bioinformatics https://david.ncifcrf.gov/home.jsp (accessed 29 April 2025). The analysis shows that in the cerebral cortex of NPC mice, the expression of genes belonging to clusters of transcriptional regulation, DNA binding, binding to metal ions, ubiquitination, and others is altered. The parameters used were an enrichment score < 1.4 and a *p* value < 0.005.

Functional classification of DEGs in the cerebral cortex of the NPC mouse compared to the WT mouse was performed with the aim of obtaining functional information of genes with a higher Z-Score value, which allowed us to identify the main biological functions altered in the NPC mouse. A Z-Score value higher than ±3.0 was used, resulting in a total of 149 genes analyzed (Supplementary Table S1), 29 overexpressed genes with a Z-Score > 3, and 120 underexpressed genes with a Z-Score < −3.0 (Figure 1). The function that affected the most genes was enzymatic activity, which accounted for 22.2% of the total number of genes, followed by differentiation and development (16.3%), signaling (10.5%), transcription (9.2%), apoptosis (7.8%), and ubiquitination (5.2%) (Figure 1A).

DEGs with a Z-Score greater than 2 and less than −2 were clustered based on annotation terms among genes using the Functional Annotation Clustering function in DAVID Bioinformatics. The parameters used were mean astringency value, enrichment score < 1.4, and *p* ≤ 0.005. The clusters with overexpressed genes were associated with binding of metal ions, lipids, zinc ions, mitochondria, and endoplasmic reticulum. The clusters with underexpressed genes were more strongly associated with transcriptional regulation, DNA repair, and ion channels. In the cluster analysis of the total number of over- or under-expressed genes, some groups of genes remained significant, such as transcriptional regulation, mitochondria, endoplasmic reticulum, differentiation, metal ion, and lipid binding genes. On the other hand, some new clusters emerged, such as elongation factor, proteosome complex, ubiquitination, and GTPase activity (Figure 1B).

### 3.2. KEGG Analysis of DEGs in the Cerebral Cortex of the NPC Mouse

KEGG pathway analysis was performed in ShinyGo (ShinyGO V0.77), Genetrail (GenetrailV3.2), and DAVID Bioinformatics using genes with a Z-Score greater than 2 and less than −2. Using these results, we created a comparative Venn diagram of the major pathways in each of the databases to determine which pathways were present in more than one of the databases used (Supplementary Figure S1). When analyzing the overexpressed genes, the estrogen signaling pathway and the ubiquitination pathways were found to be significant. Among the overexpressed genes, significant signaling pathways related to diseases caused by viruses such as hepatitis B, measles, HIV, and Epstein-Barr virus emerged, revealing unexpected links between NPC disease and some viral diseases. The ferroptosis signaling pathway appears significant in three databases and with regard to Toll-like receptors in two databases. When analyzing the total number of genes, some pathways, such as ubiquitination, estrogen signaling, hepatitis B, ferroptosis, and Toll-like receptors, remained significant, and new significant pathways were found: the Coronavirus signaling pathway and Gonadotropin-Releasing Hormone (GnRH) secretion.

### 3.3. Relevant Genes in Bioinformatics Analysis

Several important functional groups emerged from the bioinformatic analysis. However, we selected those significant gene groups in more than one analysis, i.e., functional analysis, cluster analysis, and KEGG pathway analysis. Therefore, the selected genes belong to the functional groups of ubiquitination, fatty acid metabolism, genes with mitochondrial functions, apoptosis, and development and differentiation (Table 1).

### 3.4. Validation of Microarray Expression Data by qPCR and Determination of Altered Pathways by Western Blot Protein Expression

To validate the microarray expression results, we performed qPCR expression analysis on the selected genes. The expression of the genes *Bmal1 (*Basic Helix-Loop-Helix ARNT Like 1), *Ghitm* (Growth hormone inducible transmembrane protein), *Rnf5* (Ring finger protein 5), *Ptpmt1* (Protein tyrosine phosphatase, mitochondrial 1), *Ppm1f* (Protein phosphatase 1F), *Bmp4* (Bone morphogenetic protein 4), *Bbs9* (Bardet–Biedl syndrome 9), *Fbxo45* (F-box protein 45), and *Rarg* (Retinoic acid receptor, gamma) were confirmed. On the other hand, the genes *Gpam* (Glycerol-3-phosphate acyltransferase, mitochondrial), *Palm2* (Paralemmin A kinase anchor protein), and *Slc39a1* (Solute carrier family 39 zinc transporter member 1) were found to be expressed in the same direction as in the microarray analysis, but the change in expression in qPCR was not statistically significant. The genes *Smpd1 (*Sphingomyelin phosphodiesterase 1, acid lysosomal), *Abcd1 (*ATP-binding cassette, sub-family D (ALD), member 1), *Sfxn1 (*Sideroflexin 1), *Cyp24a1 (*Cytochrome P450, family 24, subfamily a, polypeptide 1), *Uchl1* (Ubiquitin carboxy-terminal hydrolase L1), and *Ghrh* (Growth hormone releasing hormone) showed differential expression between microarray and qPCR, which could be due to the variable specificity of microarray analysis. However, the change in expression by qPCR was statistically significant and may be relevant to the phenotype in NPC.

#### 3.4.1. Expression Analysis Reveals the Dysregulation in Genes Involved in Intrinsic Apoptosis and Alterations in the 1C Metabolic Pathway in the NPC Mouse

**Figure 2:**
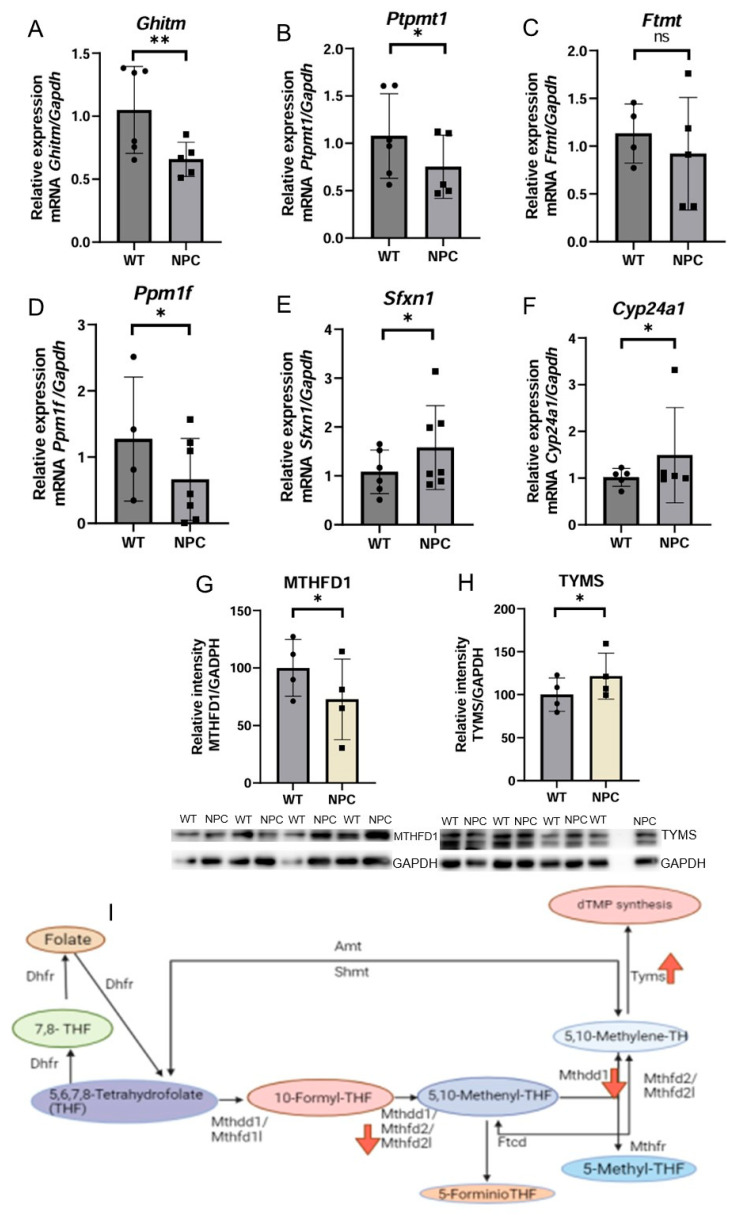
Expression of genes related to mitochondrial metabolism and apoptosis, and Western blot expression analysis of proteins involved in 1C metabolism. (**A**) The *Ghitm* gene was underexpressed in the NPC group. This result is consistent with the underexpression observed in the microarray. (**B**) The *Ptpmt1* gene was found underexpressed in the cerebral cortex of the NPC mice. (**C**) No differences in the expression of the *Ftmt* gene were found between WT and NPC mice; therefore, this gene involved in ferroptosis does not appear to be altered in the cortex of NPC mice. (**D**,**E**) Under-expression of the Ppm1f and Sfxn1 genes was observed in the cerebral cortex of NPC mice. (**F**) In contrast to the microarray results, overexpression of the gene Cyp24a1 was observed in NPC mice. (**G**) Under-expression of the MTHFD1 protein in the NPC group. (**H**) In contrast to MTHFD1 expression, overexpression of TYMS was observed. (**I**) 1C metabolic pathway; this image shows the direction of expression of the molecules involved in this pathway. Mthfd2l is underexpressed at the RNA level in the microarray. MTHFD1, the enzyme responsible for the formation of 5,10-methylene, is also underexpressed (Down red arrow). TYMS, the protein that catalyzes the synthesis of dTMP, is overexpressed (Up red arrow), probably due to a compensatory effect. *Gapdh*/GAPDH was used to normalize gene and protein expression. A *T*-Student test was performed to determine statistical significance. The normality of the data was determined using the Shapiro–Wilk normality test and the Rout outlier test to detect outliers. ** *p* ≤ 0.01, * *p* ≤ 0.05, ns: not significant.

Based on bioinformatic analysis, 6 genes whose products localize in the mitochondria were selected and are reported to exhibit either a neurological phenotype or premature death. Some of these genes have mitochondrial functions and are also involved in regulating the process of intrinsic apoptosis, *Ghitm* and *Ptpmt1*, and ferroptosis, *Ftmt.* The *Ghitm* and *Ptpmt1* genes were found to be underexpressed in NPC mice. *Cyp24a1*, which codes for the enzyme 24-hydroxylase, which is responsible for the degradation of the active form of vitamin D, was found to be overexpressed. The gene *Ppm1f* was found to be underexpressed in NPC mice (Figure 2).

The *Sfxn1* gene is underexpressed in NPC mice by qPCR. This gene has important functions in mitochondrial metabolism, such as 1C metabolism, as the transport of serine into the mitochondria is a precursor of folate synthesis. Folate is reduced to its active form, tetrahydrofolate (THF), which is the first event in 1C metabolism. Therefore, we used Western blotting to determine the expression of proteins involved in the 1C metabolic pathway (Figure 2). MTHFD1 (Methylenetetrahydrofolate Dehydrogenase, Cyclohydrolase and Formyltetrahydrofolate Synthetase 1) was found to be underexpressed in the cerebral cortex of NPC mice compared to WT mice, and the protein TYMS (thymidylate synthase) was found to be overexpressed in the cerebral cortex of NPC mice.

#### 3.4.2. Alteration in the Cortical Circadian Rhythm Pathway in the Cerebral Cortex of NPC Mice

**Figure 3:**
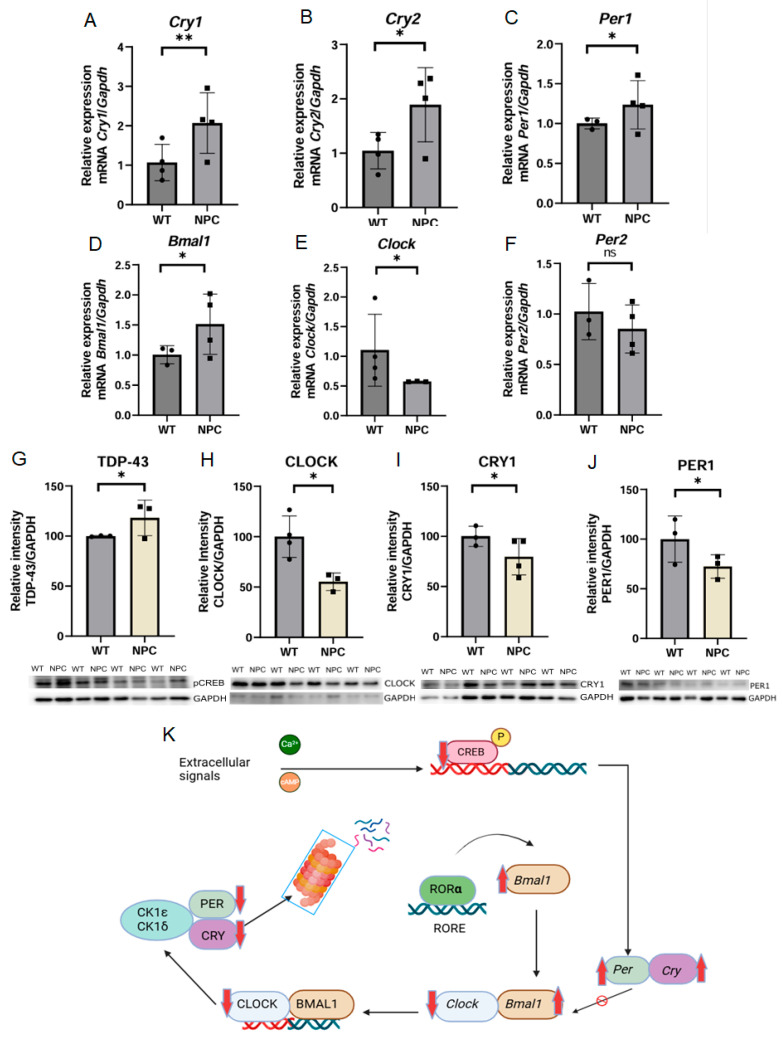
Expression of the circadian rhythm in the cerebral cortex of the NPC mouse. Results of expression analysis by qPCR of genes involved in circadian rhythm regulation. (**A**,**B**) Overexpression of the *Cry1* and *Cry2* genes, respectively, in the cerebral cortex of NPC mice compared to WT mice. (**C**,**D**) The *Per1* and *Bmal1*/*Arntl* genes were found overexpressed in the NPC group. (**E**) On the other hand, the *Clock* gene was found underexpressed in the cerebral cortex of NPC mice. (**F**) Underexpression of the *Per2* gene was observed but was not statistically significant. (**G**) The pCREB protein was found to be underexpressed in the NPC group. The ratio was determined by correlating the expression of pCREB and CREB, which was previously normalized with the expression of GAPDH protein. (**H**) Underexpression of the CLOCK protein was observed, a result consistent with the underexpression observed in qPCR. (**I**,**J**) CRY1 and PER1 proteins were found to be underexpressed in the NPC group compared to the WT group, but the change in PER1 protein expression was not statistically significant. (**K**) Summary of the results of the expression of components involved in the circadian rhythm. The pCREB protein was underexpressed, *Per1*, *Cry1*, and *Bmal1* were overexpressed at the mRNA level, in contrast to *Clock*, which was overexpressed. PER1 and CRY1 were overexpressed at the protein level while underexpression was detected at the mRNA level. Overexpression (Up red arrow), Underexpression (Down red arrow). GAPDH/*Gapdh* was used as a normalizing protein and for gene expression. A *T*-Student test was performed to determine statistical significance. The normality of the data was determined using the Shapiro–Wilk normality test and the Rout outlier test to detect outliers ** *p* ≤ 0.01, * *p* ≤ 0.05, ns: not significant.

The gene *Arntl* or *Bmal1* (Basic Helix-Loop-Helix ARNT Like 1) has been associated with apoptosis functions in Gene Ontology (GO). However, this gene is also involved in other relevant functions; one of the most important is that it encodes a transcriptional regulator of circadian rhythm by forming heterodimers with the CLOCK protein [15]. Therefore, we found it important to investigate the expression of components involved in the circadian rhythm in the cerebral cortex. First, we determined the expression of circadian rhythm components at the mRNA level and found dysregulation of the expression of key circadian rhythm components. Consistent with the microarray result, *Bmal1* was found to be overexpressed in NPC mice, while *Clock* (Clock Circadian Regulator) and Per2 (Period Circadian Regulator 2) were underexpressed, but this change in expression was not statistically significant between the groups. On the other hand, the genes *Per1* (Period Circadian Regulator 1), *Cry1* (Cryptochrome Circadian Regulator 1), and *Cry2* (Cryptochrome Circadian Regulator 2) were found to be overexpressed in the cerebral cortex of the NPC mice (Figure 3).

Subsequently, the protein levels of some components of the circadian rhythm were determined at the protein level by Western blot (Figure 3). pCREB was underexpressed in NPC mice. CRY and PER1 showed underexpression at the protein level, in contrast to what was observed for mRNA.

#### 3.4.3. Genes Associated with Ubiquitination Are Altered and Show Increased Levels of TDP-43 in the Cerebral Cortex of the NPC Mouse Mutant

**Figure 4:**
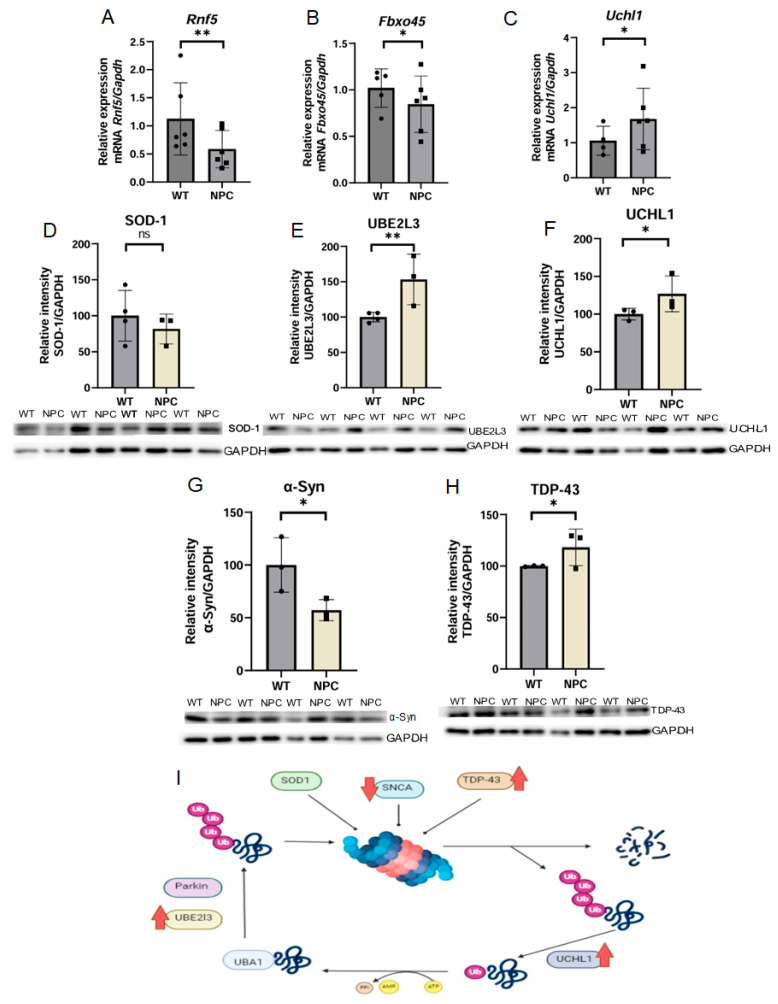
Expression of genes related to ubiquitination and Western blot expression analysis of proteins involved in ubiquitination in neurodegenerative diseases. (**A**,**B**) The genes Rnf5 and Fbxo45 were found under-expressed in the NPC mice; these results are consistent with the microarray results. (**C**) The *Uchl1* gene was found overexpressed in the cerebral cortex of the NPC group in contrast to the microarray. (**D**) Reduced levels of SOD-1 protein were observed in the cerebral cortex of NPC mice but were not statistically significant. (**E**,**F**) Increased levels of UBE2L3 and UCHL1 proteins in the cerebral cortex of NPC mice compared to WT mice. (**G**) Decreased level of α-Syn in NPC mice. (**H**) Increased levels of TDP-43 in NPC mice. (**I**) The summary of expression results of components involved in neurodegenerative diseases shows increased levels of TDP-43, UBE2L3, and UCHL1 proteins and low levels of α-Syn protein. Overexpression (Up red arrow), Underexpression (Down red arrow). GAPDH/*Gapdh* was used as a normalizing gene and in protein expression. A *T*-Student test was performed to determine statistical significance. The normality of the data was determined using the Shapiro–Wilk normality test and the Rout outlier test to detect outliers. ** *p* ≤ 0.01, * *p* ≤ 0.05, ns: not significant.

The functional analysis, gene cluster analysis, and KEGG pathway analysis results showed that genes associated with ubiquitination remained significant in all three analyses. Therefore, we selected three genes related to this process that are dysregulated in the cerebral cortex of the NPC mouse compared to the WT mouse. The *Rnf5* gene was less expressed in NPC mice compared to WT mice (Figure 4A). This gene encodes a ring finger protein with E3 ubiquitin ligase activity, which also responds to misfolded proteins associated with the endoplasmic reticulum [16]. The gene *Fbxo45* was found underexpressed in the cerebral cortex of NPC mice (Figure 4B). This gene encodes a protein with E3 ubiquitin ligase activity, which also has important functions in CNS development [17]. The *Uchl1* gene was found to be overexpressed in NPC mice by qPCR (Figure 4C). This gene encodes a protein that mediates the deubiquitination of various proteins [17].

Due to the functions of *Uchl1*, it is involved in the pathways of neurodegeneration. Therefore, the expression of proteins that are part of this pathway was determined by Western blot. The proteins analyzed were UCHL1, UBE2L3 (Ubiquitin-Conjugating Enzyme E2 L3), TDP-43, SOD-1 (Superoxide Dismutase 1), and α-Syn (α-Synuclein) (Figure 4). Increased levels of UCHL1, UBE2L3, and TDP-43 proteins and low levels of α-Syn and SOD-1 were detected, although the dysregulation of SOD-1 levels was not statistically significant.

#### 3.4.4. The Cerebral Cortex of the NPC Mouse Shows Changes in Genes Associated with Differentiation and Development

**Figure 5:**
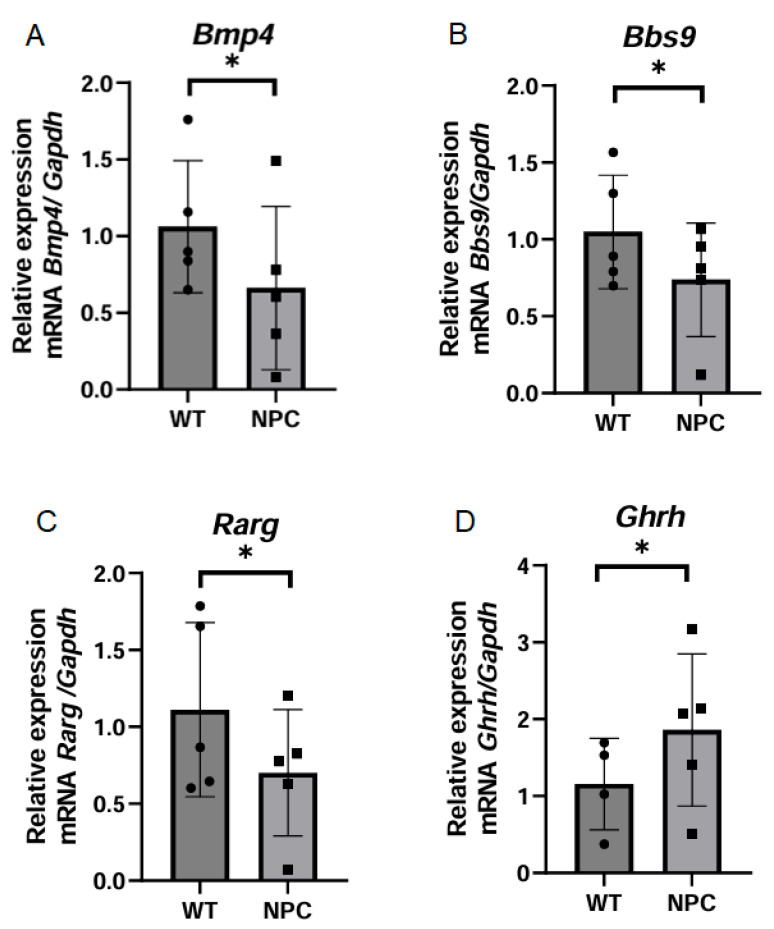
Expression of genes related to differentiation and development. (**A**–**C**) The genes Bmp4, Bbs9, and Rarg were found to be under-expressed in the cerebral cortex of the NPC group. This underexpression is consistent with that observed in the microarray. (**D**) The *Ghrh* gene was overexpressed in NPC mice. The *Gapdh* gene was used to normalize gene expression. A *T*-Student test was performed to determine statistical significance. The normality of the data was determined using the Shapiro–Wilk normality test and the Rout outlier test to detect outliers. * *p* ≤ 0.05, ns: not significant.

Seven genes with functions in differentiation and development were selected because genes associated with these functions appeared significant in the functional and cluster analysis. Based on information from MGI, there are reports on the expression and nonexpression of the gene *Pax2* in CNS tissues in adult mice; however, in our work, this gene was not expressed in the cerebral cortex of NPC mice or in WT mice. On the other hand, the *Bmp4* gene was found to be underexpressed in NPC mice (Figure 5A). This gene develops the circulatory and nervous systems [17]. Similarly, the gene *Bbs9* was found to be underexpressed in the cerebral cortex of NPC mice (Figure 5B). This gene is associated with adipocyte differentiation and cilia genesis due to its membership in the BBsome complex [17]. The *Rarg* gene, which codes for the retinoic acid receptor gamma, was found to be underexpressed in the cerebral cortex of NPC mice (Figure 5C). On the other hand, the qPCR analysis revealed that the *Ghrh* gene was overexpressed in the NPC mice (Figure 5D), which regulates sleep in the cerebral cortex [18].

#### 3.4.5. The Cerebral Cortex of NPC Mice Shows a Dysregulation of Proteins Involved in the Beta-Oxidation of VLCFA

**Figure 6:**
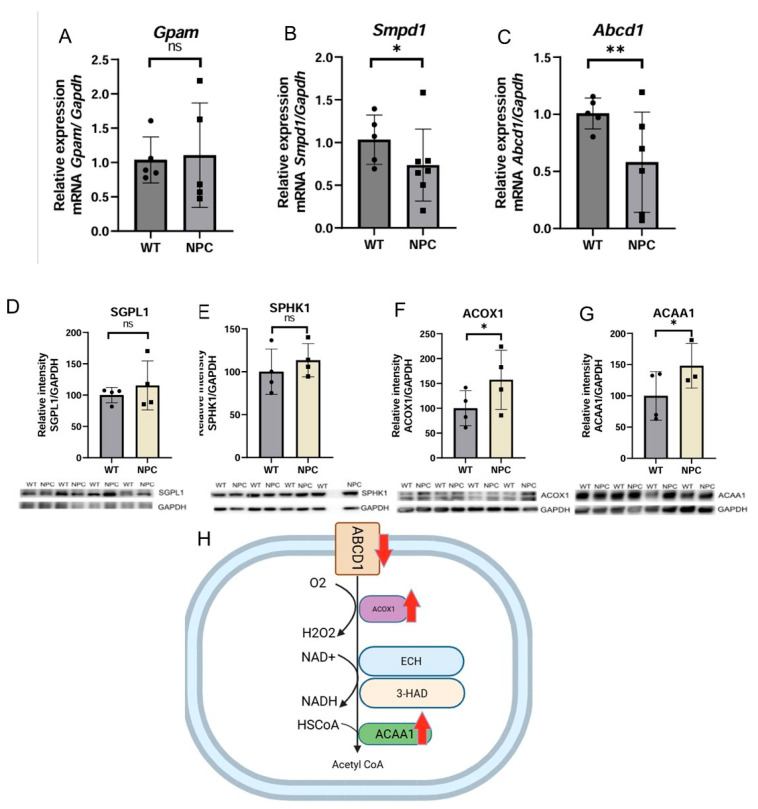
Expression of genes and proteins related to fatty acid metabolism. (**A**) No differences were detected in the expression of *Gpam* in the cerebral cortex of NPC mice. (**B**,**C**) The genes Smpd1 and Abcd1 were found under-expressed in the NPC group. (**D**,**E**) The SGPL1 and SPHK1 proteins did not differ in NPC mice from those of the WT mouse group. (**F**,**G**) Elevated levels of the proteins ACOX1 and ACAA1 were found in the cerebral cortex of NPC mice. (**H**) Altered fatty acid beta-oxidation pathway in NPC. Overexpression (Up red arrow), Underexpression (Down red arrow). *Gapdh*/GAPDH was used as a normalization gene and protein expression. A *T*-Student test was performed to determine statistical significance. The normality of data was determined using the Shapiro–Wilk normality test and the Rout outlier test to detect outliers. ** *p* ≤ 0.01, * *p* ≤ 0.05. ns: not significant.

Due to the characteristic features of NPC, genes related to fatty acid metabolism are expected to be altered, and their study is important to learn more about this disease. Therefore, genes related to fatty acid metabolism were selected. No changes in *Gpam* gene expression were detected between the group of NPC and WT (Figure 6A). The *Smpd1* gene was found to be underexpressed in the cerebral cortex of the NPC mice (Figure 6B). This gene encodes the enzyme responsible for converting sphingomyelin to ceramide, and mutations in this gene have been associated with Niemann Pick type A and B [15]. Similarly, the *Abcd1* gene was found to be underexpressed in NPC mice (Figure 6C). This gene encodes a protein responsible for transporting very long-chain fatty acids (VLCFA) to the peroxisome for the beta-oxidation process [15].

To determine whether the underexpression of the *Smpd1* gene observed in the NPC mice has an effect on the sphingolipid metabolic pathway involving the SMPD1 protein, we determined the expression of proteins downstream of the activity of this enzyme by Western blot. Increased expression of the two proteins SGPL1 (Sphingosine-1-Phosphate Lyase 1) and SPHK1 (Sphingosine Kinase 1) was observed, but in both cases, there was no statistical significance (Figure 6). Therefore, the underexpression of *Smpd1* does not appear to be important as a cause of alterations in this metabolic pathway in the cerebral cortex of NPC mice.

Based on the observed underexpression of *Abcd1* in the cerebral cortex of NPC mice, the protein expression of ACOX1 (Acyl-CoA Oxidase 1) and ACAA1 (Acetyl-CoA Acyltransferase 1) was analyzed by Western blot to determine whether there is a change in the beta-oxidation pathway of long-chain fatty acids. It was found that the ACOX1 and ACAA1 proteins were overexpressed in the cerebral cortex of NPC mice compared to WT mice (Figure 6).

## 4. Discussion

Our study reveals significant alterations in gene expression related to mitochondrial metabolism, ubiquitination, differentiation and development, fatty acid metabolism, and circadian rhythm in the cerebral cortex of NPC mice. These findings provide new insights into the molecular mechanisms underlying NPC and suggest potential therapeutic targets.

The underexpression of *Ptpmt1* and *Ghitm* in NPC mice highlights a critical disruption in mitochondrial function. These genes play roles in maintaining mitochondrial membrane integrity and regulating apoptosis. Their downregulation may contribute to the activation of intrinsic apoptosis pathways, exacerbating mitochondrial dysfunction observed in NPC.

The *Ptpmt1* gene was underexpressed in the cerebral cortex of NPC mice compared to WT mice. This gene encodes protein tyrosine phosphatase mitochondrial 1, which is involved in the dephosphorylation of phosphatidylglycerophosphate (PGP) to phosphatidylglycerol (PG), an intermediate in the biosynthesis of cardiolipin [15]. Cardiolipin is part of the mitochondrial membrane and, therefore, involved in the correct assembly of the electron transport chain complexes. Its underexpression is associated with changes in the assembly of electron chain complexes [19]. Cardiolipin gives the mitochondrial matrix a curved structure that allows it to interact with cytochrome c and hold it in place. It is well known that the release of cytochrome c is a key event in the process of intrinsic apoptosis [20,21]. The *Ghitm* gene was also found to be underexpressed in the cerebral cortex of NPC mice. This gene has important mitochondrial functions, such as preserving the morphology of the mitochondrial membrane and negative regulation of the intrinsic apoptosis pathway by mediating the interaction of cytochrome C with the mitochondrial inner membrane [22].

Previously, it has been reported that elevated cholesterol in mitochondrial membranes in NPC causes mitochondrial dysfunction and, thus, activation of intrinsic apoptosis [7]. However, the underexpression of *Ptpmt1* and *Ghitm* in the cerebral cortex found in our work may indicate a more direct activation mechanism of the intrinsic apoptosis pathway in NPC.

qPCR expression analysis revealed that the gene *Sfxn1* is underexpressed in the cerebral cortex of NPC mice. This gene encodes an integral mitochondrial membrane protein that is a mitochondrial serine transporter and has several functions, such as its involvement in 1C metabolism by transporting serine into the mitochondria, facilitating the conversion of cytosolic serine to glycine and formate in the mitochondria [23]. Formate is used to generate charged folates that serve as donors in one-carbon metabolism and enable the formation of carbon units required for the synthesis of 5,10-methylenetetrahydrofolate, a necessary substrate for purine synthesis [15,24].

Based on the result of the *Sfxn1* gene and the fact that the microarray analysis revealed an under-expression of the *Mthd2l* gene, the levels of proteins involved in the metabolism of 1C were determined to identify changes in this metabolic pathway. The proteins analyzed were MTHFD1 and TYMS (thymidylate synthase). MTHFD1 has methylenetetrahydrofolate dehydrogenase (NADP+) activity and is the enzyme responsible for the formation of 5,10-methylenetetrahydrofolate and 10-formyl-THF. Deletion of MTHFD1 leads to embryonic lethality, as its involvement in the synthesis of purines is crucial [25,26]. The TYMS protein was found overexpressed in the cerebral cortex of NPC mice. TYMS is involved in the process of dTMP biosynthesis and in the conversion of tetrahydrofolate [17]. The overexpression of this protein is likely due to underexpression of MTHD1, leading to low levels of 5,10-methylene-THF, such that overexpression of TYMS occurs as a compensatory mechanism, as previously reported by inhibition of components of the 1C signaling pathway [27,28].

In this work, we found the alteration of important genes involved in the ubiquitination process, which is one of the main mechanisms for the degradation of short-lived proteins and misfolded proteins, including proteins reported in NPC [29]. Microarray analysis revealed that the *Uchl1* gene is underexpressed, but qPCR revealed that it is overexpressed in the cerebral cortex of NPC mice. This is consistent with the reports of NPC by Cawley et al., 2023, who reported that UCHL1 levels increased with disease severity [30]. The *UCHL1* gene encodes a thiol protease enzyme with deubiquitinase function that helps to keep the monoubiquitin pool stable [15,31]. After analyzing the KEGG pathways, the UCHL1 protein was associated with the neurodegenerative disease pathway, so the levels of the proteins involved in this pathway were examined by Western blot. Elevated levels of UCHL1 and UBE2L3 proteins were found. The UBE2L3 protein is an E2 conjugation enzyme that is involved in the ubiquitination of many proteins and has been reported to be altered in Alzheimer’s disease [32,33]. An elevated level of TDP-43 was found in the cerebral cortex of NPC mice. Dardis et al. (2016) analyzed TDP-43 levels in the cerebellum of a mouse model and found low concentrations of TDP-43 protein in the nucleus and higher accumulation in the cytosol, suggesting mislocalization in cerebellum [34]. In our study, the total amount of TDP-43 was examined, so the ratio between the nuclear and cytosolic protein is not known, but we can conclude that there is an accumulation of total TDP-43 in the cerebral cortex of NPC mice. In a postmortem study in a patient with NPC, the accumulation of α-Syn was found in the frontotemporal region [35], but in our study, only small amounts of α-Syn were found in the cerebral cortex of NPC mice. Regarding SOD-1 protein, no changes were detected in the cerebral cortex of NPC mice compared to WT mice, which is consistent with the levels reported by Ribas et al. (2012) in the plasma of NPC patients [36].

The *Rnf5* gene was found to be underexpressed in the cerebral cortex of NPC mice. This gene encodes an E3 ubiquitin ligase protein but is also involved in the ERAD pathway in response to unfolded proteins (UPR) [37], so its deregulation could lead to changes in the processing of proteins regulated by *Rnf5* in the ERAD pathway. Another gene with ubiquitination functions that is underexpressed in the cerebral cortex of mice is *Fbxo45* gene, which encodes an ubiquitin ligase and has important functions in the CNS, contributing to synapses and neuronal development [38]. Its importance is reflected in KO models of *Fbxo45*, in which alterations at the synapses of neuromuscular junctions and abnormal development of axonal fiber tracts have been observed [39].

Interestingly, genes like *Abcd1*, associated with X-linked adrenoleukodystrophy, and *Bbs9*, linked to Bardet–Biedl syndrome, show underexpression in NPC mice. These findings suggest that NPC shares molecular mechanisms with other neurodegenerative diseases, potentially opening avenues for cross-disease therapeutic strategies.

The *Abcd1* gene is involved in fatty acid metabolism as it encodes a protein that is involved in the elimination of very long chain fatty acids (VLCFA) by mediating their transport to the peroxisome [15]. Underexpression of ABCD1 has already been identified in X-linked adrenoleukodystrophy (X-ALD). In this pathology, there is an accumulation of VLCFA, and there are two main clinical forms: the cerebral type (CALD) and adrenomyeloneuropathy (AMN). In CALD, neuroinflammation, demyelination, and behavioral and cognitive deficits occur. In AMN, axonal degeneration occurs with clumsy gait and leg weakness [40,41]. Increased cholesterol levels have also been observed in Abcd1-deficient mouse models [42], so we can observe some similarities with the NPC phenotype, raising the possibility that Abcd1 underexpression may be related to some neurological symptoms of NPC. Due to the involvement of *Abcd1* in the beta-oxidation pathway of VLCFA, the protein expression of ACOX1 and ACAA1, which are involved in this pathway, was examined; the levels of ACOX1 and ACAA1 were elevated. ACOX1 is an enzyme involved in the first and rate-limiting step of the pathway, and ACAA1 catalyzes the thiolysis of 3-ketoacyl-CoAs. It can therefore be said that the underexpression of *Abcd1* could lead to an accumulation of VLCFA, which in turn leads to an increase in the levels of the enzymes ACAA1 and ACOX1, which regulate VLCFA levels. Another gene associated with diseases with a neurological phenotype is the *Bbs9* gene, which was underexpressed in the cerebral cortex of NPC mice. This gene encodes a protein that is part of the BBsome complex, which is involved in protein transport to the cilia and ciliogenesis [15,17]. Underexpression of this gene has been associated with Bardet–Biedl syndrome, which causes retinopathy, renal abnormalities, obesity, mental retardation, gait abnormalities, and CNS-related ataxia [43,44]. The latter neurological symptoms have also been reported in NPC, so the underexpression of this gene may be relevant to the occurrence of neurological symptoms in NPC.

Regarding the analyzed genes with functions in differentiation and development, in the expression analysis, we found the gene *Bmp4* underexpressed in the cerebral cortex of NPC mice. This gene encodes bone morphogenetic protein 4, a member of the TGF-β superfamily, and this protein is involved in osteogenesis and the development of neurons and glial cells at embryonic, postnatal, and postinjury stages [15,45]. In addition, mutations in this gene have been identified in about 1% of cases as a rare cause of syndromic microphthalmos, a disease that causes retinal dystrophy, myopia, and brain abnormalities [46]. The *Rarg* gene was also underexpressed in the cerebral cortex of NPC mice compared to WT mice. This gene encodes the retinoic acid receptor gamma, which acts as a transcriptional regulator by forming heterodimers with retinoic acid response elements, and is involved in embryonic morphogenesis [15,17]. *Rarg* is localized in the nucleus to regulate transcriptional activity and has higher expression in neural stem cells that are not yet differentiated, so its expression is likely to be lower in already differentiated neuronal cells, as well as in the process of neurite outgrowth [47].

The *Arntl* or *Bmal1* gene was found overexpressed in the cerebral cortex of NPC mice compared to WT mice. This gene encodes the BMAL1 protein, which regulates the circadian rhythm by forming heterodimers with the CLOCK protein [15]. This gene is expressed in cerebral cortex neurons, where it regulates the cortical circadian cycle [48,49]. Therefore, we thought it was important to assess the expression of components involved in the cortical circadian rhythm to determine whether there are changes in the cerebral cortex of NPC mice. In this analysis, we found that the pCREB protein was underexpressed in NPC mice. Phosphorylation of CREB is a key event in the signaling of melatonin biosynthesis [50]. Melatonin levels are reduced during the day, but here, we can observe a greater reduction in its expression in NPC mice compared to WT mice. The CLOCK underexpression was consistent with that observed at the mRNA level. The underexpression of CRY and PER1 at the protein level contrasts with what was observed at the mRNA level. However, this makes sense as the components of the circadian rhythm constitute a feedback loop and self-regulate their expression. Therefore, *Cry* and *Per1* are likely overexpressed at the mRNA level while they are underexpressed at the protein level [51]. As we can observe, the cortical circadian rhythm is generally disrupted in NPC mice. However, the functions of this dysregulation are less known than those of the master circadian regulator expressed in the suprachiasmatic nucleus of the hypothalamus, but it has been observed that deletion of Bmal1 leads to depressive behavior [52]. In addition, decreased melatonin levels also promote the development of depression, supporting the theory that alteration of this pathway may lead to the development of depressive behavior in NPC [53].

The underexpression of the gene *Ppm1f* is also associated with the development of depressive behavior, and its overexpression causes an antidepressant effect, possibly by modulating the expression of *Bdnf* (Brain-Derived Neurotrophic Factor) [54]. This gene was underexpressed in both microarray and qPCR expression analysis in NPC mice, so it may also be involved in the development of depressive disorders observed in some NPC patients.

While prior studies have noted elevated inflammatory markers in the brains of NPC mice and patients, no groups of inflammatory genes were found among the differentially expressed genes (DEGs) in our current study. This apparent inconsistency could be due to several reasons, among others, for the specific brain region analyzed, the disease stage at the time of sampling, or possible differences in the detection sensitivity of the different methods. Further investigations are needed to clarify these differences.

## 5. Conclusions

The study of altered metabolic pathways and gene expression in NPC is a first step toward identifying potential areas for future research. While the discovery of new genes associated with this pathology is valuable, much remains to be done to fully understand their role. The genes and signaling pathways highlighted in this study, such as those associated with ubiquitination, mitochondrial metabolism, and components of the circadian rhythm, are initial indications of potential targets for investigation. These findings suggest that dysregulations in these areas may contribute to NPC symptoms, including the behavioral disturbances associated with circadian rhythm disorders. However, the potential for developing therapeutic strategies—such as targeted ubiquitination, modulation of mitochondrial function, or stabilization of circadian rhythm—remains speculative at this stage. Sustained research efforts could contribute to novel interventions in the long term, particularly to address the associated complications, although significant challenges and uncertainties remain. In conclusion, our study elucidates key molecular alterations in NPC and highlights potential therapeutic targets. The connections between NPC and other neurodegenerative diseases underscore the importance of continued research into these shared mechanisms for developing effective treatments.

Expression analysis of the cerebral cortex of NPC mouse mutants revealed a tendency to underexpress genes. Through bioinformatic analysis, we identified groups of genes altered in NPC that are involved in important functions, such as ubiquitination, mitochondrial metabolism, apoptosis, differentiation and development, and fatty acid metabolism. Likewise, in this work we found alterations in metabolic pathways not previously reported in NPC, such as 1C metabolism, beta-oxidation of VLCFA, cortical circadian rhythm, and ubiquitination in neurodegenerative diseases. On the other hand, altered genes were found in the cerebral cortex, such as *Ptpmt1*, *Bmp4*, *Rnf5*, *Rarg*, *Ghitm*, and *Arntl*, opening the door to possible new disease biomarkers. Moreover, this study detected alterations in the *Abcd1*, *Smpd1* and *Bbs9* genes, which also play a role in other neurodegenerative diseases, suggesting links between NPC and other pathologies with similar characteristics. In the future, it will be of interest to determine for some of these genes which cell types differentially express them. Finally, this study allows us to elucidate new molecular mechanisms altered in NPC mice and identify new potential therapeutic targets.

## Figures and Tables

**Table 1:** Summary of gene groups found to be dysregulated in bioinformatics analysis.

Functional Analysis	Cluster Analysis	KEGG Pathway Analysis
Enzyme activity	Transcription regulation	Ubiquitination
Differentiation	Differentiation	Estrogen signaling
Transcription	Mitochondria	Ferroptosis
Apoptosis	Ubiquitination	GnRH secretion
Ubiquitination	Lipid binding	Toll-like receptors
Fatty acid metabolism	Proteosome complex	

## Data Availability

The data presented in this study are available on request from the corresponding author.

## Supplementary Materials

The following supporting information can be downloaded at https://www.mdpi.com/article/10.3390/genes16080865/s1: Figure S1: Comparison of the KEGG pathway analyzes; Table S1: Differentially expressed genes (DEGs) in the Cerebral Cortex of the NPC Mouse Mutant.

## Abbreviations

The following abbreviations are used in this manuscript:

ABCD1	ATP-binding cassette, sub-family D (ALD), member 1
ACAA1	Acetyl-CoA Acyltransferase 1
ACOX1	Acyl-CoA Oxidase 1
BBS9	Bardet–Biedl syndrome 9
BDNF	Brain-Derived Neurotrophic Factor
BMAL1	Basic Helix-Loop-Helix ARNT Like 1
BMP4	Bone morphogenetic protein 4
cDNA	Complementary DNA
CNS	Central Nervous System
CREB	CAMP Responsive Element Binding Protein
CRY1	Cryptochrome Circadian Regulator 1
CRY2	Cryptochrome Circadian Regulator 2
Ct	Cycling threshold
CYP24A1	Cytochrome P450, family 24, subfamily a, polypeptide 1
DEGs	Differentially expressed genes
dUTP	Deoxyuridine Triphosphate
FBS	Fetal bovine serum
FBXO45	F-box protein 45
GAPDH	Glyceraldehyde-3-phosphate dehydrogenase
GHITM	Growth hormone inducible transmembrane protein
GHRH	Growth hormone releasing hormone
GnRH	Gonadotropin-Releasing Hormone
GPAM	Glycerol-3-phosphate acyltransferase, mitochondrial
HIV	Human immunodeficiency virus
KEGG	Kyoto Encyclopedia of Genes and Genomes
LSD	Lysosomal Storage Diseases
MGI	Mouse Genome Informatics
MHFDL2	Methylenetetrahydrofolate Dehydrogenase NADP+ Dependent 2 Like
mRNA	Messenger ribonucleic acid
MTHD1	Methylenetetrahydrofolate Dehydrogenase, Cyclohydrolase and Formyltetrahydrofolate Synthetase 1
NPC	Niemann Pick type C
NPC1	NPC Intracellular Cholesterol Transporter 1 protein
NPC2	NPC Intracellular Cholesterol Transporter 2 protein
PER1	Period Circadian Regulator 1
PPM1F	Protein phosphatase 1F
PVDF	Polyvinylidene difluoride
qPCR	Quantitative polymerase chain reaction
RARG	Retinoic acid receptor, gamma
RNA	Ribonucleic acid
RNF5	Ring finger protein 5
SEM	Standard error of the mean
SFXN1	Sideroflexin 1
SGPL1	Sphingosine- 1-Phosphate Lyase 1
SLC39A1	Solute carrier family 39 zinc transporter member 1
SMPD1	Sphingomyelin phosphodiesterase 1, acid lysosomal
SOD- 1	Superoxide Dismutase 1
SPHK1	Sphingosine Kinase 1
TBS	Tris-buffered saline
TDP-43	TAR DNA-binding protein 43
TYMS	Thymidylate synthase
UBE2L3	Ubiquitin Conjugating Enzyme E2 L3
UCHL1	Ubiquitin carboxy-terminal hydrolase L1
VLCFA	Very Long Chain Fatty Acids
WT	Wild type
α-Syn	α-synuclein
PGP	Phosphatidylglycerophosphate
PG	Phosphatidylglycerol
X-ALD	X-linked adrenoleukodystrophy
CALD	Cerebral adrenoleukodystrophy
AMN	Adrenomyeloneuropathy
