# Supplementary material for: Gene Expression Profile of the Cerebral Cortex of Niemann-Pick Disease Type C Mutant Mice
Source: Genes (Basel). 2025 Jul 24;16(8):865. doi: 10.3390/genes16080865 (PMC12386176; doi:10.3390/genes16080865)
Supplement: Supplementary file 1 [file genes-16-00865-s001.zip › genes-3737733-Supplementary Figure S1.pdf]

Supplementary Figure S1

Gene Expression Profile of the Cerebral Cortex of Niemann-Pick Disease Type C Mutant Mice

Iris Valeria Servín-Muñoz [1,2,†], Daniel Ortuño-Sahagún [1,*,†], María Paulina Reyes-Mata [3], Christian Griñán-Ferré [4,5], Mercè Pallàs [4,5] and Celia González-Castillo [6,*]

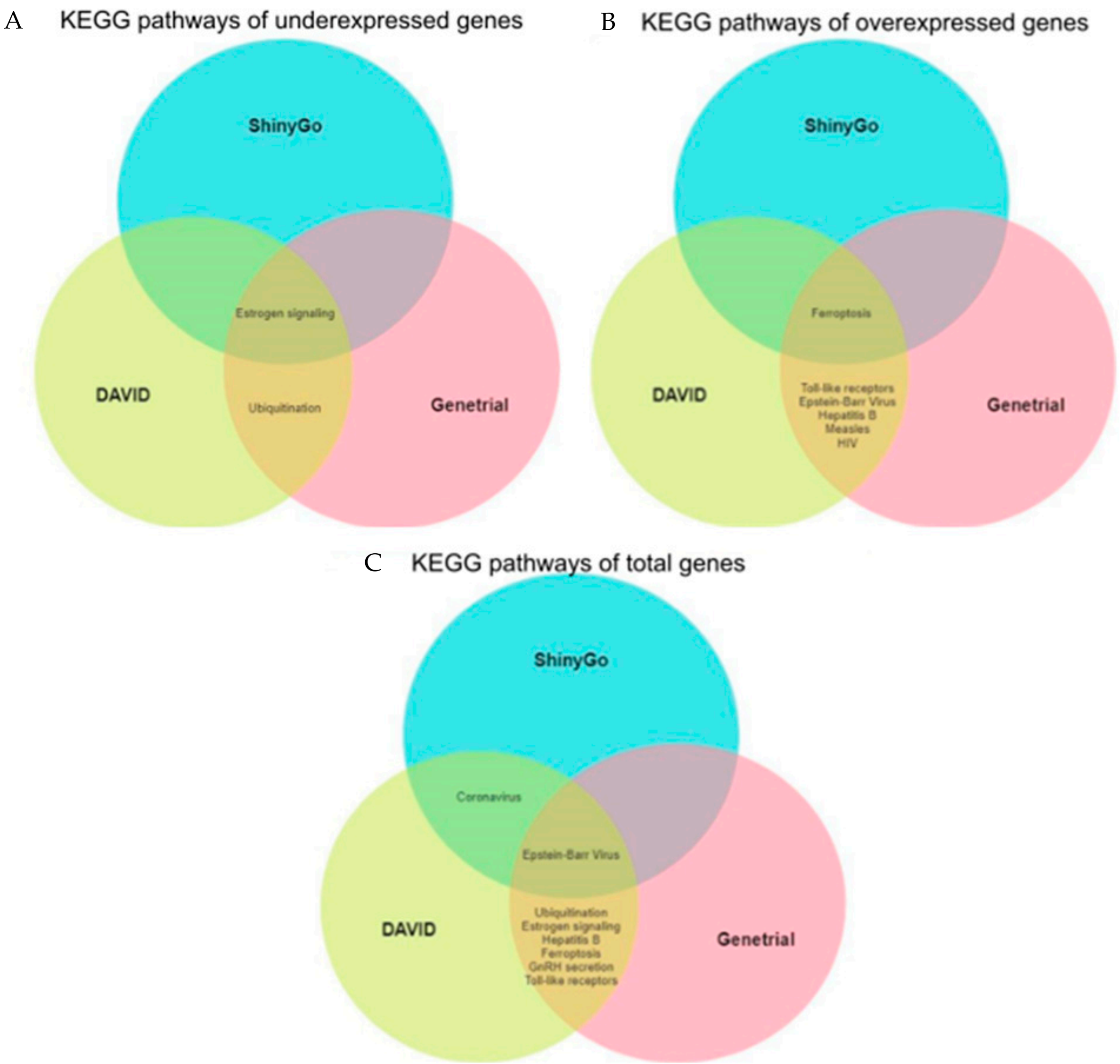

Supplementary Figure S1. Comparison of the KEGG pathway analyzes. (A) Comparison of the KEGG pathway results of the underexpressed genes with a Z-Score < 2. It can be seen that in all three databases the estrogen signaling pathway appears significant and in two of these databases the ubiquitination pathway appears as significant. (B) Comparison of the results of the KEGG pathways for overexpressed genes with a Z-Score > 2. The common deregulated pathway in the three databases is the ferroptosis pathway. (C) The ubiquitination pathway is again significant when comparing the three databases with the total DEGs. The analysis of the KEGG pathways was performed in the databases ShinyGo (ShinyGO V0.77), Genetrail (Genetrail V3.2) and DAVID bioinformatics https:/david.ncifcrf.gov/home.jsp (accessed 29 April 2024).
